# Tuft cell cysteinyl leukotrienes are necessary for rhinovirus-induced mucus metaplasia, type 2 inflammation and airway hyperresponsiveness in immature mice

**DOI:** 10.3389/fimmu.2025.1709008

**Published:** 2026-01-07

**Authors:** De’Jana T. Parker, J. Kelley Bentley, Jing Lei, Baljeet Domala, Hannah L. Briggs, Shilpi Singh, Yiran Li, M. Claire Reiner, Derek A. Flores, Alex L. Sliwicki, Heidi R. Flori, Marc B. Hershenson

**Affiliations:** Department of Pediatrics, University of Michigan Medical School, Ann Arbor, MI, United States

**Keywords:** asthma, cysteinyl leukotriene, respiratory viral, rhinovirus, tuft cells

## Abstract

**Introduction:**

Early-life wheezing-associated respiratory tract infections with rhinovirus (RV) are considered risk factors for asthma development. Cysteinyl leukotrienes (cysLTs) are pro-inflammatory lipid mediators synthesized from arachidonic acid by 5-lipoxygenase (Alox5) and Alox5 activating protein (Alox5ap). We hypothesized that tuft cell-derived cysLTs are required for the development of an asthma phenotype in immature mice undergoing heterotypic RV infection.

**Methods:**

We infected C57BL/6, *Alox5*-/- or *Pou2f3*-/- mice lacking tuft cells with sham HeLa cell lysate or RV-A1B on day 6 of life and RV-A2 on day 13 of life. Selected mice were treated with montelukast or vehicle. Lungs were harvested on day 21 for ELISA, histology, immunohistochemistry, immunofluorescence microscopy and qPCR. Airways responsiveness to methacholine was determined by plethysmography. We also examined nasal swabs from children hospitalized with RV bronchiolitis for *ALOX5* and *ALOX5AP* transcripts.

**Results:**

After heterologous RV infection, C57BL/6 mice showed increased lung cysLT levels and mRNA expression of *Alox5* and *Alox5ap*. *ALOX5* and *ALOX5AP* were also increased in infants with RV bronchiolitis. RV-infected mice demonstrated rare Alox5+, Alox5ap+ and Dclk1+ cells in the airway epithelium, indicating the presence of tuft cells. RV-infected *Pou2f3*-/- mice showed reduced lung cysLT production and an absence of Alox5+, Alox5ap+ or Dclk1+ epithelial cells. *Alox5*-/- and *Pou2f3*-/- mice, as well as montelukast-treated C57Bl/6 mice, showed reduced Muc5ac levels, PAS staining, airways responsiveness and mRNA expression of *Il4*, *Il5*, *Il13* and *Il25*.

**Conclusions:**

Tuft cell-derived cysLTs are required for mucous metaplasia, type 2 inflammation and airways hyperresponsiveness in immature mice exposed to heterologous viral infection.

## Introduction

Early-life wheezing-associated respiratory tract infections by rhinovirus (RV) and respiratory syncytial virus (RSV) are considered risk factors for asthma development. Birth cohort studies from Tucson (1), Wisconsin (2–4), Western Australia (5), Perth (6) and the Netherlands (7) have shown associations between wheezing lower respiratory tract illness and asthma in children up to 13 years of age. In the Wisconsin cohort, early life wheezing with RV, but not RSV, was associated with asthma development (2–4). These data suggest that early life respiratory viral infection, in particular with RV, contributes to the development of asthma, in some cases in the absence of allergen sensitization. Alternatively, it is conceivable that early life wheezing with RV simply identifies children who are already destined to develop asthma.

To test whether early-life viral infection contributes to asthma development, we established a mouse model of early-life RV infection. RV infection of 6 day-old mice, but not mature mice, induces type 2 airway inflammation, mucous metaplasia and airways hyperresponsiveness (8) which is associated with expansion of IL-13-producing type 2 innate lymphoid cells (ILC2s) and dependent on the innate cytokines IL-25, IL-33 and TSLP (9, 10). Subsequent studies showed that ILC2s are required and sufficient for the asthma-like phenotype (11) and that IFN-γ blocks the phenotype by interfering with ILC2 function (12). Early-life RV infection alters the response to subsequent heterologous infection, inducing intensified mucous metaplasia and airway hyperresponsiveness which are dependent on ILC2s (13). Finally, we found that heterologous RV infection increases the number of airway tuft cells, and that tuft cells are required for the asthma phenotype (14).

Tuft cells are rare chemosensory epithelial cells that monitor their environment and relay messages to surrounding tissue via secretion of neuromodulatory and immunomodulatory molecules (15, 16). In the airway, tuft cells express elements of the bitter taste transduction system including the Tas2R receptors α-gustducin and TRPM5 (17), a calcium-activated non-selective cation channel. Nearly all tuft cells express ChAT (choline acetyltransferase). All tuft cells share a common developmental identity driven by the transcription factor Pou2f3, which is necessary for their generation (18–20). A subset of tuft cells plays an immunomodulatory function. In mice, tuft cell-specific deletion of leukotriene C4 synthase (Ltc4s), the terminal enzyme required cysteinyl leukotriene (cysLT) production, reduces *Alternaria*-induced ILC2 expansion and lung inflammation (21). Like innate cytokines, cysLTs drive ILC2 activation (22–24). Aeroallergen-induced expansion of tuft cells is also dependent on Ltc4s (25), indicating an autocrine role for cysLTs. Together, these data suggest that cysLTs, produced by airway tuft cells, promote ILC2 activation and type 2 inflammation.

The cysLTs are synthesized from arachidonic acid by arachidonate 5-lipoxygenase (ALOX5). ALOX5 converts arachidonic acid into 5-hydroperoxyeicosatetraenoic acid (5-HPETE), which spontaneously reduces to 5-hydroxyeicosatetraenoic acid (5-HETE). ALOX5 acts again on 5-HETE to convert it into leukotriene A4 (LTA4), an unstable epoxide. ALOX5 activating protein (ALOX5AP) acts as a membrane anchor and is required for ALOX5 activation. In cells that express LTC4, LTA4 is conjugated with the tripeptide glutathione to form the first of the cysLTs, LTC4. Outside the cell, LTC4 can be converted by ubiquitous enzymes to form successively LTD4 and LTE4. In the present study, we employed *Alox5* and *Pou2f3* null mice to examine the contribution of tuft cell-derived cysLTs in the development of mucous metaplasia, type 2 inflammation and airways hypersponsiveness following early-life heterologous RV infection.

## Methods

### Animals

C57Bl/6 and *Alox5*-/- mice (B6.129S2-*Alox5^tm1Fun^*/J) (26) were purchased from Jackson Laboratories (Bar Harbor, ME). *Skn-1a/Pou2f3*-/- mice were provided by Dr.Ichiro Matsumoto (18). Mice were bred in-house in a pathogen-free facility within the Unit for Laboratory Animal Medicine at the University of Michigan. All animal usage was approved by the Institutional Animal Care and Use Committee and followed guidelines set forth in the Principles of Laboratory Animal Care from the National Society for Medical Research.

### Viruses and viral preparation

RV-A1B and RV-A2 (ATCC, Manassas,VA) are minor group RVs that infect mouse epithelial cells (27). Viruses were partially purified and concentrated from infected HeLa cell lysates by ultrafiltration using a 100 kDa cutoff filter (28).

Similarly concentrated and purified HeLa cell lysates were used for sham infection. Viruses were quantitated by plaque assay. Concentrated virus stocks were maintained at >10^8^ plaque forming units (PFU)/mL.

### Infection of mice

C57Bl/6, *Alox5*-/- or *Pou2f3*-/- mice were inoculated with 15 μL sham HeLa cell lysate or RV-A1B (1 x 10^6^ PFU) on day 6 of life and HeLa cell lysate or RV-A2 (1x10^6^ PFU) on day 13 of life. Both sexes of mice were used in this study, as no significant differences in the response to heterologous infection were observed (**Supplementary Material Figure**).

### Montelukast treatment

Selected C57Bl/6 mice were infected with RV-A1B and RV-A2 as described above and treated intranasally with vehicle or montelukast sodium (Cayman Chemical, Ann Arbor, MI) on days 6–20 of life. Montelukast was dissolved in Tris-buffered saline, pH 8.5, at a concentration of 10 µg/µl. We administered 10 µg montelukast per g body weight. The rationale for using intranasal administration was that it is easier and safer than intraperitoneal administration or gavage in baby mice. Intranasal administration of RV, vehicle and montelukast was carried out under brief isoflurane anesthesia.

### Measurement of lung cysLTs

C57Bl/6, *Alox5* null or *Pou2f3* KO mice were inoculated with heterologous RV as described above. Mice were euthanized via CO2 chamber and death was verified with cervical dislocation. Mice were perfused through the right ventricle with phosphate buffered saline (PBS), 5 mM EDTA, complete protease inhibitors (Sigma Chemical, St. Louis, MO) and 10 μM indomethacin (Cayman Chemical, Ann Arbor, MI). Perfused lungs were placed in 1 mL of PBS + EDTA, snap frozen in liquid nitrogen, and thawed with homogenization until completely blended. The homogenate was then centrifuged at 13,000 x *g* at 4°C for 10 min to obtain a clarified supernatant fluid. Supernatant mouse IgG was removed with agarose-immobilized protein G (Sigma). Supernatant proteins were precipitated by dilution into four volumes cold ethanol and centrifuged for 10 min at 13,000 x *g*. The 80% ethanol-soluble fraction was nitrogen-purged and vacuum dried under nitrogen with no heat. The dried pellets were resuspended in Cayman’s 0.05% polysorbate 80 assay buffer and processed with dilution for measurement of total cysLTs by ELISA (Cayman Chemical).

### Lung histology

After perfusion (described above), lungs were inflated with formalin, fixed in 10% formalin overnight, and processed for paraffin embedding and sectioning. Five-micron paraffin sections were processed for periodic acid-Schiff stains of mucins, immunofluorescence and immunohistochemistry. For periodic acid-Schiff (Sigma Chemical) staining, deparaffinized sections were stained according to manufacturer’s instructions, counterstained with Gill’s hematoxylin, dehydrated and mounted. Images were visualized using an Olympus IX71 microscope with appropriate filters. NIH ImageJ software was used to quantitate PAS staining in the epithelium. An equidistant 8 x 11 point grid was overlaid on a 4x images of lung sections, and PAS abundance calculated as the fraction of points over PAS-positive epithelium compared with the total number of points on the grid.

For immunofluorescence staining, sections were permeabilized with Tris-buffered saline with Tween-20 (TBST) + 1% Triton X-100, blocked with 10% goat serum and 5% bovine serum albumin in TBST, and incubated with 1 μg/mL rabbit anti-Alox5ap), anti-Dclk1 (both from Abcam, Waltham, MA) or rabbit IgG (Sigma) in blocking buffer overnight at 4°C. After washes in TBST, AlexaFluor 488 or 594-labeled goat anti-rabbit (InVitrogen, Waltham, MA) was used at a 1:1000 dilution to localize primary antibody binding. Nuclei were stained with 4′,6-diamidino-2-phenylindole (DAPI).

### Measurement of cytokine mRNA by quantitative PCR

After perfusion, lungs were either snap frozen in 1 mL Trizol (InVitrogen) according to manufacturer’s instructions. The aqueous phase post-extraction was diluted in an equal volume ethanol and processed for total RNA purification using RNeasy spin columns (Qiagen, Germantown, MD). After Nanodrop spectral quantitation and purity assessment, first strand cDNA was prepared using random hexamers with a Multiscribe reverse transcription kit (InVitrogen). Subsequent qPCR was performed using specific primers (**Table 1**) and expression relative to GAPDH was calculated using the 2–ΔΔCt method.

**Table 1:** Primer sequences for real-time PCR.

Mouse *Alox5* (forward)	5′-TCTTCCTGGCACGACTTTGCTG-3′
Mouse *Alox5* (reverse)	5′-GCAGCCATTCAGGAACTGGTAG-3′
Mouse *Alox5ap* (forward)	5’-TTTGAGCGGGTCTACACTGC-3’
Mouse *Alox5ap* (reverse)	5’-GTCCTGCAGTCCAGAGTACC-3’
Mouse *Clca1* (forward)	5′-CTGTCTTCCTCTTGATCCTCCA-3′
Mouse *Clca1* (reverse)	5′-CGTGGTCTATGGCGATGACG-3′
Mouse *Gapdh* (forward)	5′-GTCGGTGTGAACGGATTTG-3′
Mouse *Gapdh* (reverse)	5′-GTCGTTGATGGCAACAATCTC-3′
Mouse *Il4* (forward)	5’-GGTCTCAACCCCCAGCTAGT-3’
Mouse *Il4* (reverse)	5’-GCCGATGATCTCTCTCAAGTGAT-3’
Mouse *Il5* (forward)	5′-CTCTGTTGACAAGCAATGAGACG-3′
Mouse *Il5* (reverse)	5′-TCTTCAGTATGTCTAGCCCCTG-3′
Mouse *Il13* (forward)	5′-CCTGGCTCTTGCTTGCCTT-3′
Mouse *Il13* (reverse)	5′-GGTCTTGTGTGATGTTGCTCA-3′
Mouse *Il25* (forward)	5′-ACAGGGACTTGAATCGGGTC-3′
Mouse *Il25* (reverse)	5′-TGGTAAAGTGGGACGGAGTTG-3′
Mouse *Il33* (forward)	5′-GGCTGCATGCCAACGACAAGG-3′
Mouse *Il33* (reverse)	5′-AAGGCCTGTTCCGGAGGCGA-3′
Mouse *Muc5ac* (forward)	5′-AAAGACACCAGTAGTCACTCAGCAA-3′
Mouse *Muc5ac* (reverse)	5′-GGTTTGACACTGACTTCCCAG-3′
Mouse *Pou2f3* (forward)	5’-GTTCGCCAAGACCTTCAAGCAG-3’
Mouse *Pou2f3* (reverse)	5’-GCGAGATGGTAGTCTGGCTGAA-3’
Mouse *Tslp* (forward)	5′-CCAGGCTACCCTGAAACTGA-3′
Mouse *Tslp* (reverse)	5′-TCTGGAGATTGCATGAAGGA-3′

### Measurement of Muc5ac and cytokine proteins by ELISA

Mouse lungs were harvested in PBS + EDTA with protease inhibitors and indomethacin, snap frozen in liquid nitrogen and thawed with homogenization until completely blended as described above. The supernatant fluid was diluted 1:10 in TBST. Total protein was measured by BCA protein assay (ThermoFisher Scientific). Mouse Muc5ac, TSLP and IL-17E/IL-25 ELISA kits were purchased from InVitrogen. All other ELISA immunoassays were performed using stock assays at the Immune Monitoring Core of the Michigan Rogel Cancer Center.

### Measurement of airways responsiveness

Mice were anesthetized with ketamine and pentobarbital, tracheotomized and examined for airways resistance using a Buxco FinePointe operating system (Buxco, Wilmington, NC), as described (8). Airway responsiveness was assessed by measuring changes in total respiratory system resistance in response to increasing doses of nebulized methacholine.

### Analysis of nasal swabs for *Alox5* and *Alox5ap* transcripts

This study was approved by the University of Michigan Medical School Institutional Review Board (ID# HUM00133414). We had the opportunity to analyze *Alox5* and *Alox5ap* transcripts as part of larger study examining the effects of respiratory viral infections on upper respiratory tract gene expression in hospitalized children. Nasal swabs from 17 children with RV bronchiolitis were compared with samples from six uninfected children undergoing elective otolaryngologic procedures (**Table 2**). Samples were taken from the surface of the nasal inferior turbinate using a Copan floxed swab (Murrieta, CA), placed in RNAlater (ThermoFisher Scientific, Waltham, MA), and brought to the laboratory for processing and analysis.

**Table 2:** Description of patient samples.

ID	Diagnosis	Age (mo)	Race/ethnicity	Sex
1126	bronchiolitis	9	white	male
1124	bronchiolitis	6	mixed	female
1324	bronchiolitis	19	white	female
2101	bronchiolitis	11	white	male
2308	bronchiolitis	17	Hispanic	male
2309	bronchiolitis	21	white	female
2104	bronchiolitis	10	black	female
2107	bronchiolitis	11	Asian	female
1331	bronchiolitis	17	Asian	male
1327	bronchiolitis	11	black	male
2110	bronchiolitis	8	white	male
2105	bronchiolitis	11	white	male
2320	bronchiolitis	21	mixed	female
2324	bronchiolitis	21	black	male
2327	bronchiolitis	18	white	male
1136	bronchiolitis	10	mixed	male
2334	bronchiolitis	18	white	male
2807	control	192	white	male
2410	control	44	mixed	male
2612	control	99	white	male
2609	control	114	white	female
2806	control	182	white	male
2805	control	200	white	male

Total RNA was isolated from the whole cell pellets using TRIzol-LS (ThermoFisher) extraction and purified using an RNeasy Mini Kit (Qiagen, Germantown, MD) with DNase incubation. Measurement of RNA concentration and RIN (RNA integrity number), library generation and next generation sequencing were performed by the University of Michigan Advanced Genomic Core. Samples were enriched for mRNA using poly(A) selection for low-input cDNA library preparation and subsequently underwent 150 bp paired-end sequencing using Illumina HiSeq technology (SanDiego, CA). Sequence reads were trimmed using Trimmomatic v0.36 and mapped to the Homo sapiens GRCh38 reference genome using the STAR aligner v2.5.2b (29, 30). Unique gene hit counts were calculated using featureCounts from the Subread v1.5.2 package (31, 32). Differential gene expression analysis was performed using R package DESeq2 v1.46.0 (33) with default parameters. Data were adjusted for age using limma:removeBatchEffect (34).

### Statistical analysis

When comparing group differences, statistical significance was assessed by the Mann-Whitney test or Kruskal-Wallis test with correction for multiple comparisons by the two-stage linear step-up procedure of Benjamini, Krieger, and Yekutieli.

## Results

### Heterologous RV infection stimulates cysLT production and mRNA expression of *Alox5* and *Alox5ap*

We infected C57Bl/6, *Alox5*-/- and *Pou2f3*-/- mice with HeLa cell lysate or RV-A1B on day 6 of life and HeLa cell lysate or RV-A2 on day 13 of life. The day 6 time point was chosen because mice at this age have an immature, type 2-biased response to viral infection, unlike more mature mice (9). We infected the mice with a second virus on day 21 to increase this response (13). We used two different strains because children are infected with many different RV strains, with infants having 6–10 distinct RV infections per year (35). There are more than 160 antigenically distinct strains of RV and therefore children rarely are re-infected with same strain. RV-A1 and RV-A2 do not induce specific immunity to reinfection (36).

**Figure 1:**
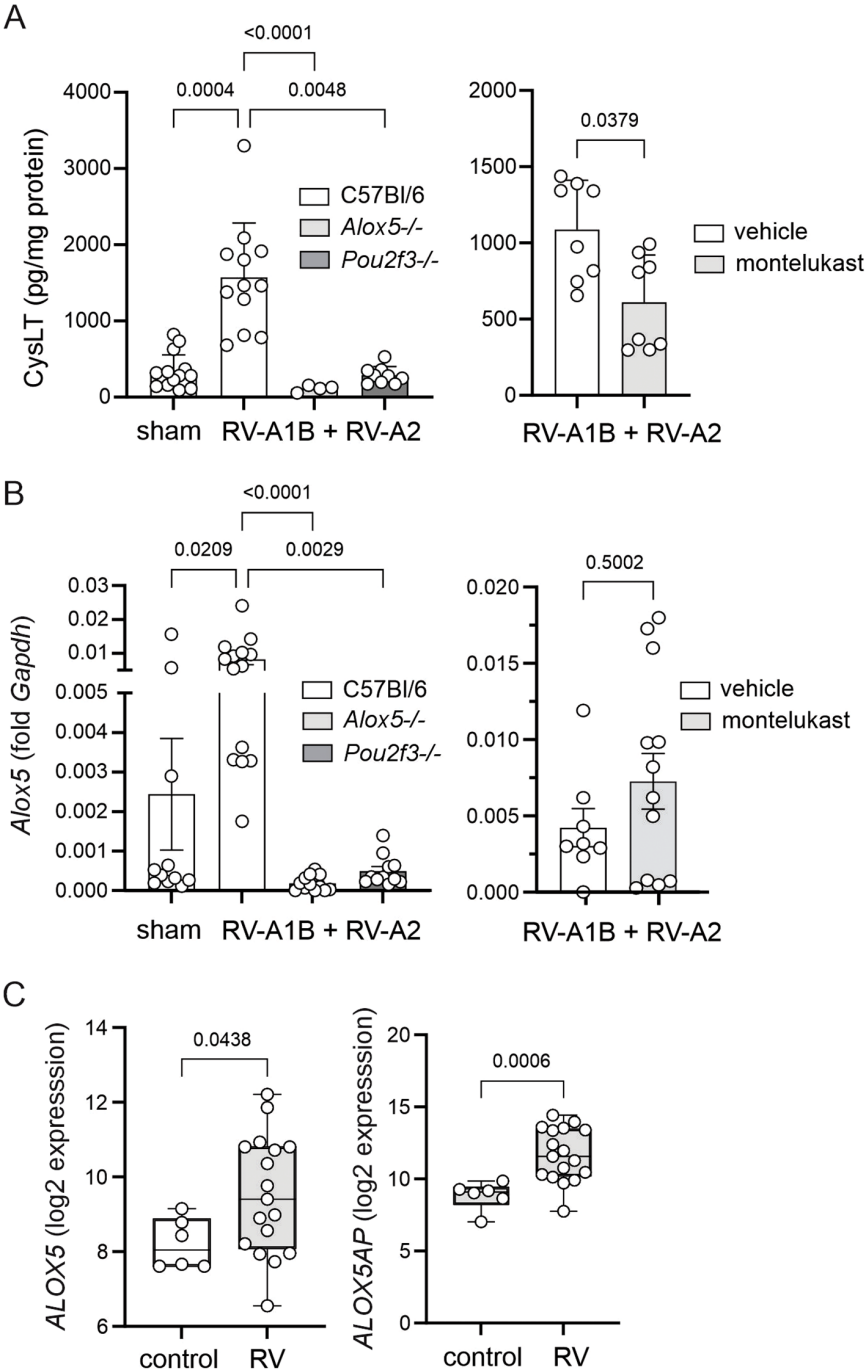
RV treatment stimulates lung cysLT levels in C57BL/6 mice. Total lung cysLTs were measured by ELISA. **(A)** Left panel: CysLT levels in sham and RV-infected C57Bl/6, *Alox5*-/- and *Pou2f3*-/- mice. (Mean ± SD, n=4–15 per group, 1–6 experiments per condition). Right panel: CysLT levels in vehicle and montelukast-treated RV-infected C57Bl/6 mice. (Mean ± SD, n=4 per group, 1 experiment per condition). **(B)** Left panel: *Alox5* mRNA expression in sham and RV-infected 57Bl/6, Alox5-/- and Pou2f3-/- mice. (Mean ± SD, n=6–12 per group, 2–3 experiments per condition.) Right panel: *Alox5* mRNA expression in vehicle and montelukast-treated RV-infected C57Bl/6 mice. (Mean ± SD, n=7 per group, 2 experiments per condition.) For A and B, data were analyzed by Kruskal-Wallis test or Wilcoxon matched paired signed rank test. Dunn’s multiple comparisons test was used to pinpoint differences identified by the Kruskal-Wallis test. **(C)***ALOX5* and *ALOX5AP* transcripts in nasal samples from healthy children and infants with RV bronchiolitis. Shown are median, interquartile range (forming the box), and whiskers extending to the minimum and maximum values. Wald test p values were adjusted for multiple testing using the procedure of Benjamini and Hochberg.

In order to assess the cysLT response to heterologous viral infection, we measured the levels of cysLTs from lungs of infected C57Bl/6 mice by ELISA. Compared to sham-treated mice, C57Bl/6 mice treated with RV-A1B on day 6 of life and RV-A2 on day 13 of life showed significantly increased lung cysLT levels (**Figure 1A**). Heterologous RV infection also significantly increased mRNA expression of *Alox5* (**Figure 1B**). RV infection had no significant effect on mRNA expression of Alox5ap (not shown).

### Measurement of human *Alox5* and *Alox5ap* expression in nasal aspirates from infants with RV infections

We also had the opportunity to analyze *ALOX5* and *ALOX5AP* transcripts as part of larger study examining the effects of respiratory viral infections on upper respiratory tract gene expression in hospitalized children. Nasal swabs from 17 children with RV bronchiolitis were compared with samples from six uninfected children undergoing elective otolaryngologic procedures. RNA transcripts were measured by next generation sequencing and differences in gene expression calculated using DESeq2 (33). Data were adjusted for age using limma:removeBatchEffect (34). Expression of *ALOX5* and *ALOX5AP* was significantly increased in nasal swabs from infants with RV infection compared to control subjects (**Figure 1C**), consistent with a role of cysLTs in the response to early life RV infection.

### Requirements of *Alox5* and *Pou2f3* for cysLT production

We measured the effects of *Alox5* and *Pou2f3* knockouts on cysLT protein levels. RV-infected *Pou2f3*-/- mice lacking tuft cells showed reduced RV-induced cysLT production, suggesting that heterologous RV infection induces cysLT production from airway tuft cells (**Figure 1A**). *Alox5-/-* mice failed to show any increase in cysLT levels after RV infection, consistent with the requirement of Alox5 for cysLT production. Finally, compared to vehicle-treated mice, montelukast-treated mice showed a partial decrease in cysLT production (**Figure 1A**), suggesting that cysLT production after heterologous RV infection is dependent in part on a positive cysLT feedback loop. Montelukast had no effect on levels of *Alox5* (**Figure 1B**) or *Alox5ap* (not shown).

### Localization of *Alox5* and *Alox5p* protein in mouse lungs from RV-infected mice

**Figure 2:**
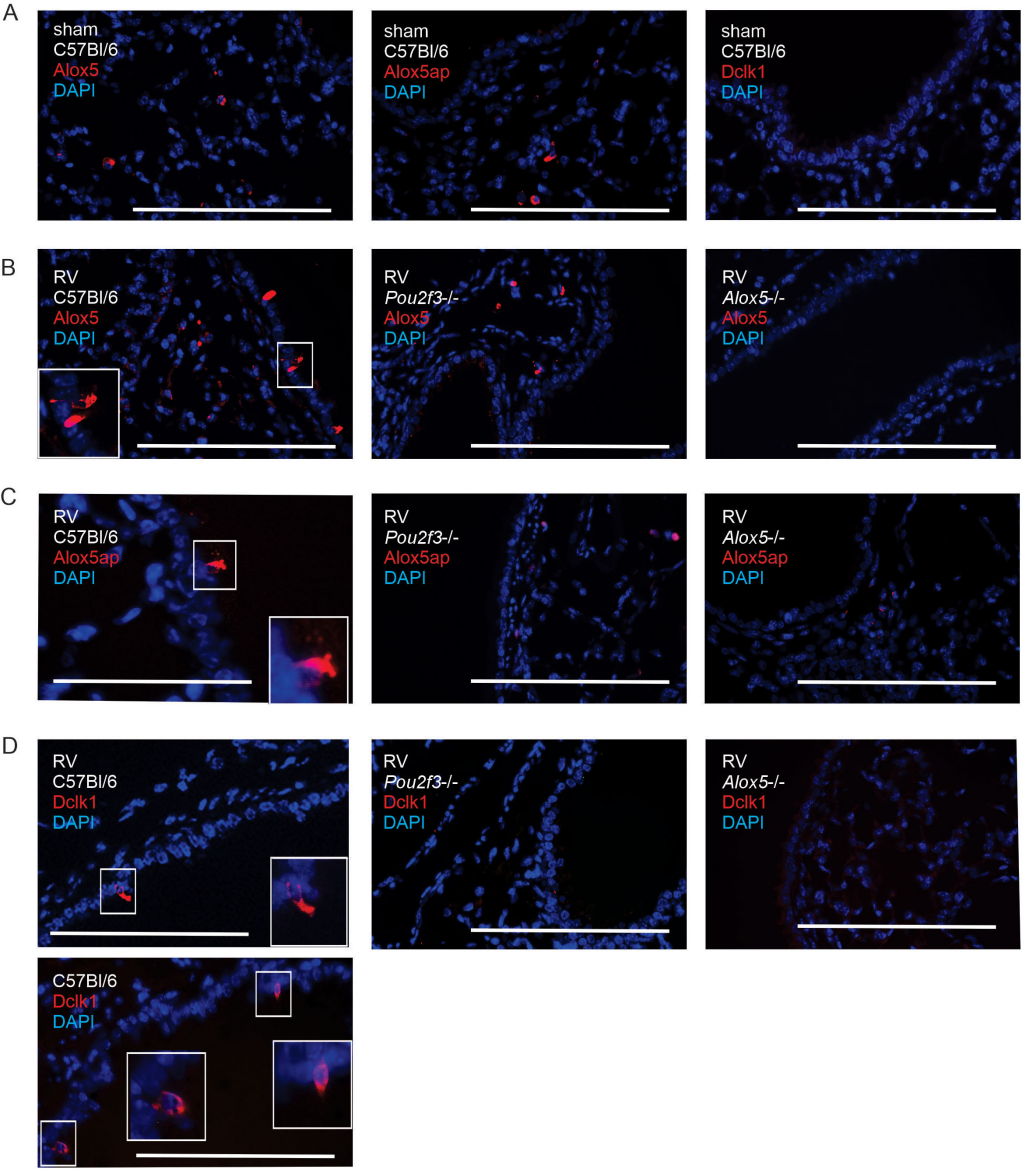
Localization of Alox5, Alox5ap and Dclk1 in airway epithelium. **(A)** Immunofluorescent staining for Alox5, Alox5ap and Dclk1 in uninfected wild-type C57Bl/6 mice. Lungs from uninfected wild-type mice show no Dclk1 staining in the airway epithelium and round Alox5+ cells were present in the subepithelium. The white bars are 100 microns. **(B-D)**. Immunofluorescence staining for Alox5 **(B)**, Alox5ap **(C)** and Dclk1 [**(D)**, each shown in red]. For **(B-D)**, RV-infected mice show rare triangular or bottle-like shaped cells, a morphology typical of tuft cells. There was no staining in *Pou2f*3-/- mice (middle panels) or *Alox5-/-* mice (right panels). DAPI-stained nuclei are shown in blue. The white bars are 100 microns.

*Alox5ap* and doublecortin-like kinase-1 (*Dclk1*) are highly expressed in mouse tracheal tuft cells (15). *Alox5* and *Alox5ap* are expressed in human airway tuft cells (37) and mouse gastrointestinal tuft cells (38, 39). We previously found that heterologous RV infection increases the number of airway Dclk1+ tuft cells (14). We therefore immunostained lungs from sham and RV-infected wild-type, *Alox5*-/- and *Pou2f3*-/- mice for Alox5ap, Alox5 and Dclk1. Lungs from uninfected wild-type mice showed no Dclk1 staining in the airway epithelium, indicating an absence of tuft cells (**Figure 2A**). Lungs from RV-infected wild-type mice, but not sham-treated mice, showed rare Alox5+, Alox5ap+ and Dclk1+ cells in the airway epithelium with a triangular or bottle-like shape, typical of tuft cells (**Figures 2B–D**). Alox5ap+, Alox5+ or Dclk1+ epithelial cells were absent in *Pou2f3*-/- mice (**Figures 2B–D**, middle panels). Round Alox5+ cells were present in the subepithelium of C57Bl/6 mice and *Pou2f3*-/- mice (**Figures 2A–C**). Together, these results suggest that airway tuft cells expressing Alox5 and Alox5ap are induced by heterologous RV infection. Alox5ap+ cells were also absent in *Alox5*-/- mice (**Figures 2B–D**, right panels), consistent with the notion that tuft cell expansion is dependent on CysLTs.

### Effects of *Alox5-/-* and *Pou2f3-/-* on RV-induced mucous metaplasia

**Figure 3:**
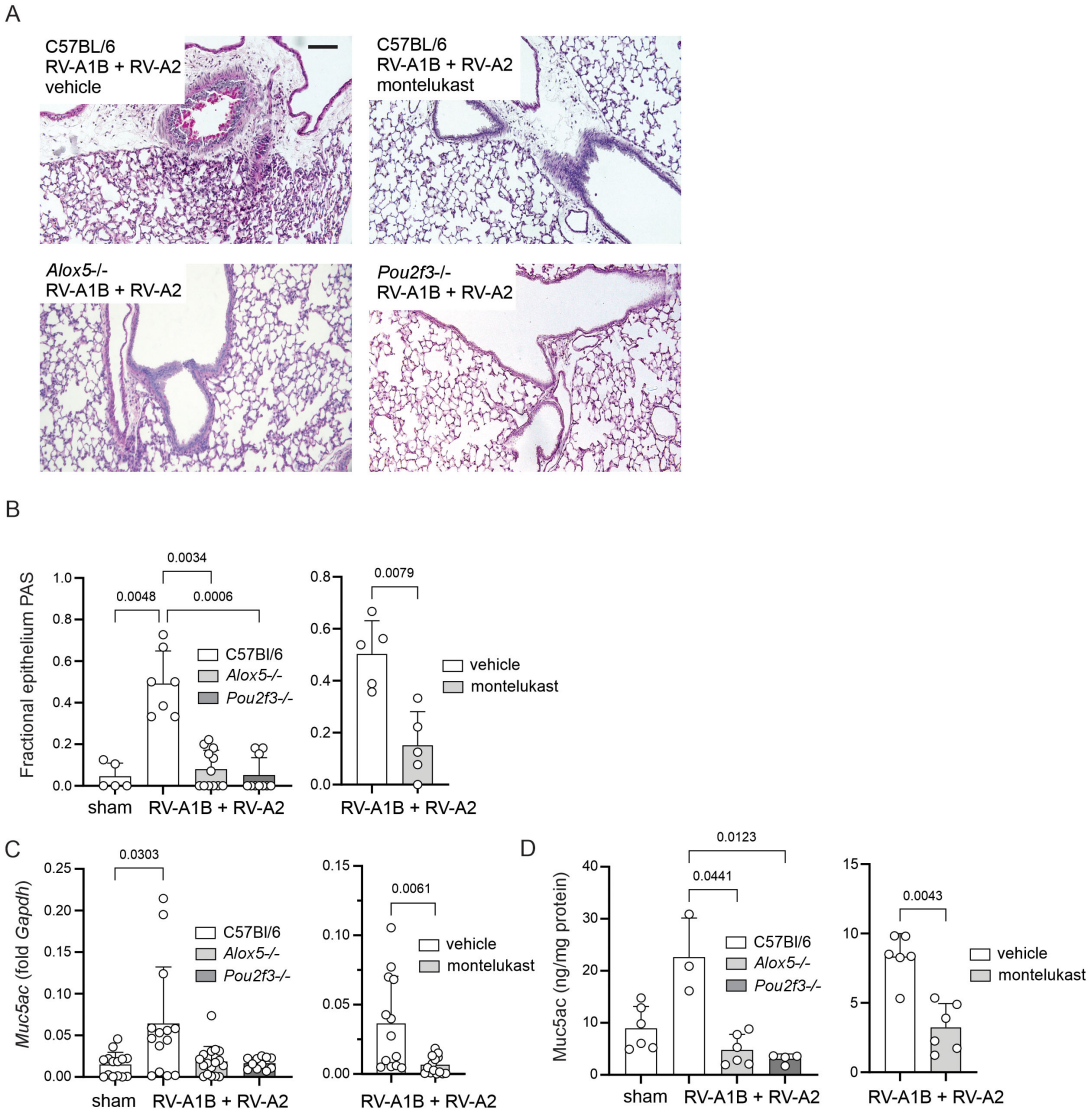
Heterologous RV infection induces mucous metaplasia in the lungs of C57Bl/6 mice but not *Alox5-/-* or *Pou2f3-/-* mice. **(A)** Lungs were harvested on day of life 20, processed for histology and stained with periodic acid-Schiff (PAS) and counterstained with hematoxylin. Heterologous RV infection increased PAS staining (magenta). Montelukast-treated C57Bl/6, *Alox5-/-* and *Pou2f3-/-* mice showed reduced PAS staining. The black bar is 200 microns. **(B)** PAS staining was quantified by point counting using NIH ImageJ. Heterologous RV infection increased the fraction of epithelia stained with PAS. Left panel: Fraction of epithelium stained with PAS in sham and RV-infected C57Bl/6, Alox5-/- and Pou2f3-/- mice. (Mean ± SD, n=4–8 per group, 2–3 experiments per condition.) Right panel: Fraction of epithelium stained with PAS in vehicle and montelukast-treated RV-infected C57Bl/6 mice. (Mean ± SD, n=5 per group, 2 experiments per condition.) **(C)** Muc5ac mRNA levels measured by qPCR. Left panel: *Muc5ac* mRNA expression in sham and RV-infected C57Bl/6, Alox5-/- and Pou2f3-/- mice. (Mean ± SD, n=11-17, 3–4 experiments per condition.) Right panel: *Muc5ac* mRNA expression in vehicle and montelukast-treated RV-infected C57Bl/6 mice. (Mean ± SD, n=4, 1 experiment per condition). **(D)** Muc5ac protein levels measured by ELISA. Left panel: Muc5ac protein expression in sham and RV-infected C57Bl/6, Alox5-/- and Pou2f3-/- mice. (Mean ± SD, n=3–6 per group, 1–2 experiments per condition.) Right panel: Muc5ac protein expression in vehicle and montelukast-treated RV-infected C57Bl/6 mice. (Mean ± SD, n=6 per group, 2 experiments per condition.) For B-D, data were analyzed by Kruskal-Wallis test or Wilcoxon matched paired signed rank test. Dunn’s multiple comparisons test was used to pinpoint differences identified by the Kruskal-Wallis test.

Compared to sham-treated mice, wild-type mice treated with RV-A1B on day 6 of life and RV-A2 on day 13 of life showed mucous metaplasia, as evidenced by PAS staining (**Figure 3A**). PAS staining was quantified by point counting (**Figure 3B**). RV infection also increased lung Muc5ac mRNA expression and protein abundance, as measured by qPCR and ELISA (**Figure 3C**). Compared to RV-treated wild type mice, *Alox5-/- and Pou2f3*-/- showed reduced PAS staining and Muc5ac mRNA and protein expression. Finally, compared to RV-infected, vehicle-treated mice, montelukast also blocked PAS staining and Muc5ac expression (**Figures 3B, C**).

### Effect of *Alox5-/-* and *Pou2f3-/-* on lung cytokine mRNA and protein expression

**Figure 4:**
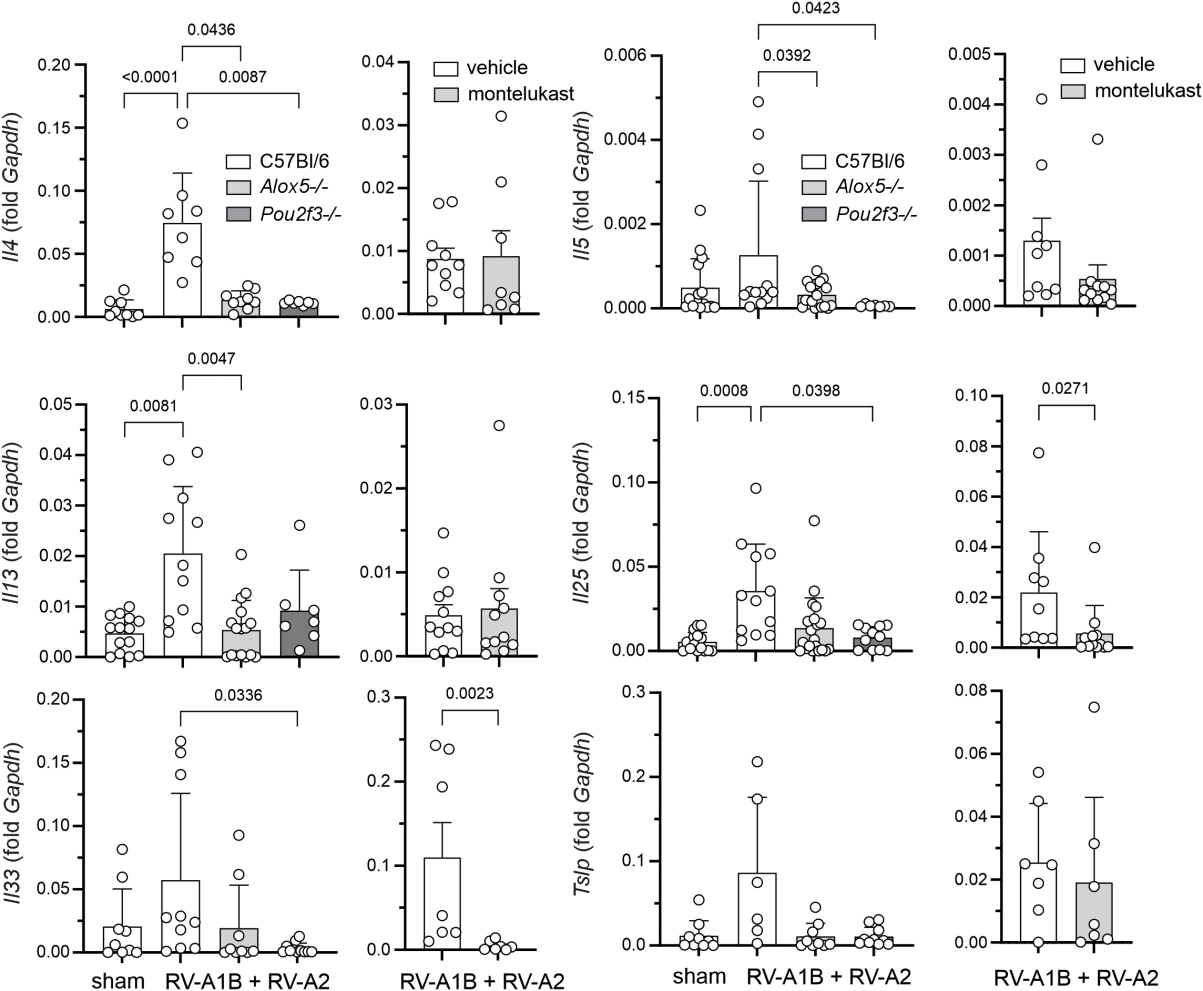
Cytokine mRNA expression in RV-infected mice. Lungs were harvested on day 20 and processed for mRNA expression. Left panels: mRNA expression in sham and RV-infected C57Bl/6, Alox5-/- and Pou2f3-/- mice. (Mean ± SD, n=7-21, 2–4 experiments per condition.) Right panels: mRNA expression in vehicle and montelukast-treated RV-infected C57Bl/6 mice. (Mean ± SD, n=7-12, 2–4 experiments per condition.) Data were analyzed by Kruskal-Wallis test or Wilcoxon matched paired signed rank test. Dunn’s multiple comparisons test was used to pinpoint differences identified by the Kruskal-Wallis test.

**Figure 5:**
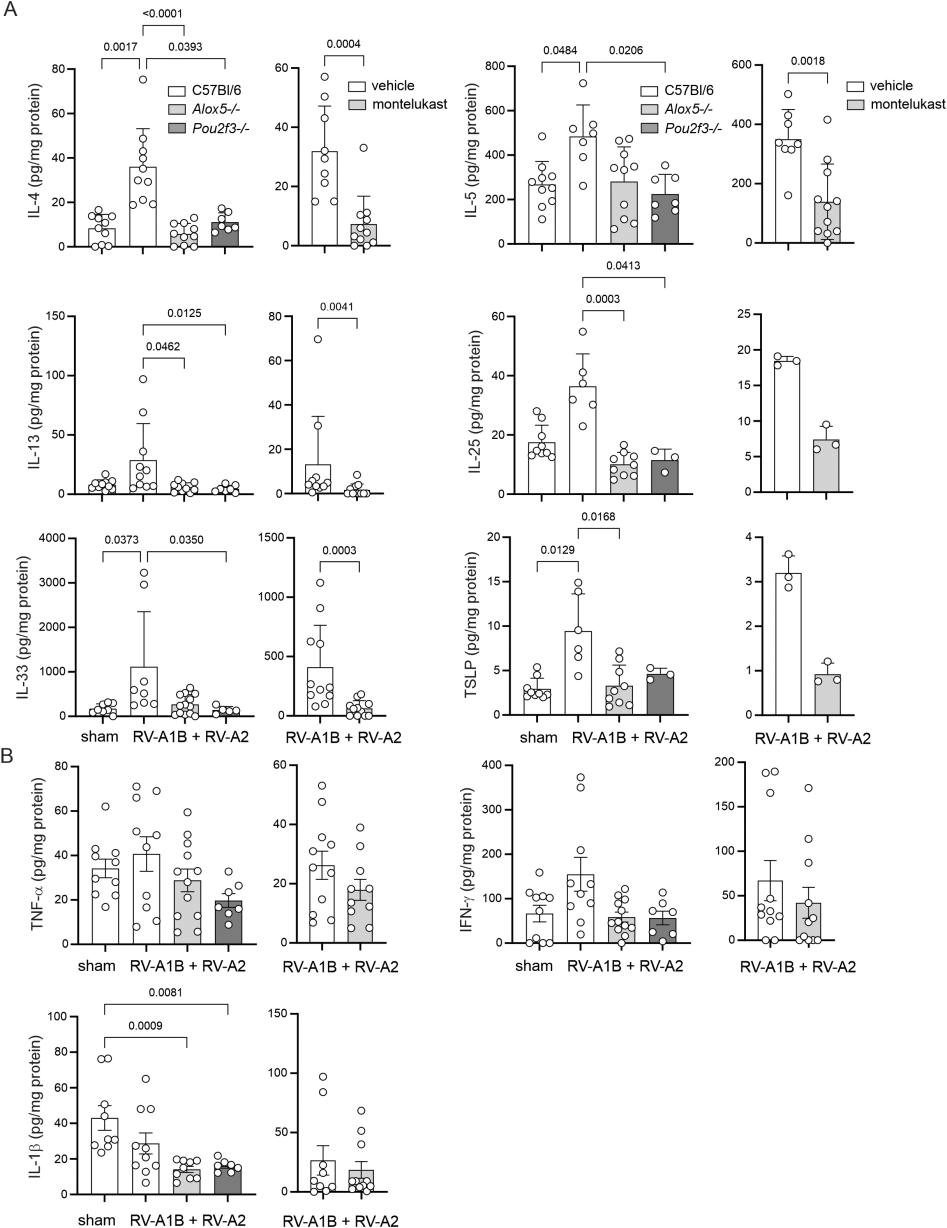
Cytokine protein expression in RV-infected mice. Lungs were harvested on day 20 and processed for protein expression by ELISA. **(A)** Type 2 and innate cytokines. **(B)** Type 1 cytokines. Left panels: Protein expression in sham and RV-infected C57Bl/6, *Alox5*-/- and *Pou2f3*-/- mice. (Mean ± SD, n=3–16 per group, 1–4 experiments per condition.) Right panels: Protein expression in vehicle and montelukast-treated RV-infected C57Bl/6 mice. (Mean ± SD, n=3–11 per group, 1–3 experiments per condition.) Data were analyzed by Kruskal-Wallis test or Wilcoxon matched paired signed rank test. Dunn’s multiple comparisons test was used to pinpoint differences identified by the Kruskal-Wallis test.

Mice exposed to RV-A1B on day 6 of life and RV-A2 on day 13 of life show a type 2 inflammatory response. Heterologous infection of wild-type mice significantly increased mRNA expression of *Il4, Il5, Il13* (**Figure 4**). RV infection also increased expression of the innate cytokines *Il25, Il33* and *Tslp*. Induction of type 2 and innate cytokine mRNA expression was blocked in *Alox5-/-* and *Pou2f3*-/- mice. Similar results were obtained for type 2 and innate cytokine protein levels (**Figure 5A**). Heterotypic RV infection did not significantly increase type 1 cytokine protein expression (**Figure 5B**). Compared to RV-infected, vehicle-treated mice, montelukast also tended to reduce type 2 cytokine mRNA and protein expression (**Figures 4**, **5A**).

### Effects of *Alox5-/-* and *Pou2f3-/-* on RV-induced airways hyperresponsiveness

**Figure 6:**
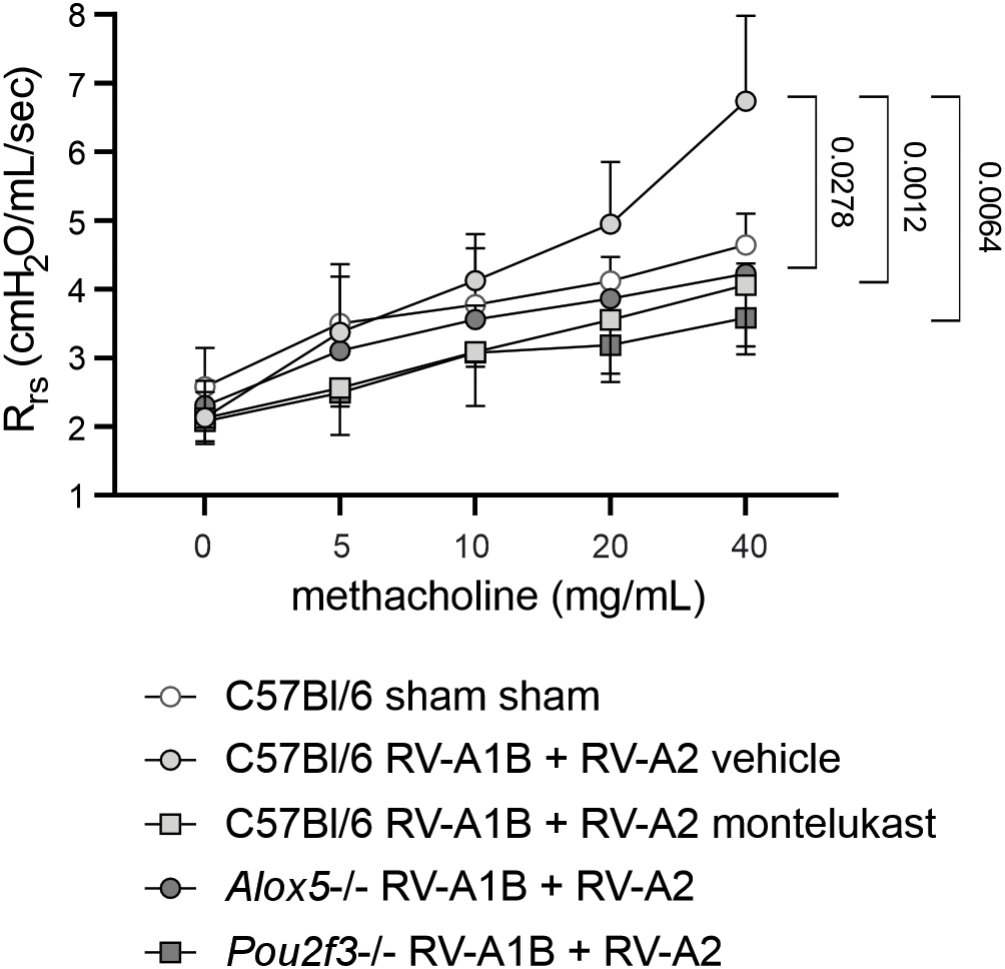
RV-induced airways responsiveness is blocked in *Alox5-/-* and *Pou2f3-/-* mice and montelukast-treated C57Bl/6 mice. Airway responsiveness was assessed by measuring changes in total respiratory system resistance in response to increasing doses of nebulized methacholine. (Mean ± SD, n=5–10 for each condition from two separate experiments.) Total respiratory system resistance (Rrs) data at 40 mg/mL methacholine were analyzed by Kruskal-Wallis test. Dunn’s multiple comparisons test was used to pinpoint differences identified by the Kruskal-Wallis test.

Compared to sham-treated mice, mice treated with RV-A1B on day 6 of life and RV-A2 on day 13 of life showed increased airways responsiveness to 40 mg/mL aerosolized methacholine on day 21 of life (**Figure 6**). Montelukast and knockout of *Alox5* and *Pou2f3* each significantly lowered maximal airways resistance.

## Discussion

Early-life wheezing-associated respiratory tract infections with RV are considered risk factors for asthma development. To test whether early-life viral infection contributes to asthma development, we created a mouse model of early-life RV infection. RV infection of 6 day-old mice, but not mature mice, induces type airway inflammation, mucous metaplasia and airways hyperresponsiveness which is associated with expansion of IL-13-producing ILC2s and dependent on the innate cytokines IL-25, IL-33 and TSLP (9, 10). Like innate cytokines, cysLTs drive ILC2 activation (22–24). Tuft cells generate cysLTs in response to aeroallergens (21). We therefore hypothesized that, in immature mice undergoing heterologous RV infection, airway tuft cells produce cysLTs. *Alox5ap* is highly expressed in mouse tracheal tuft cells (15). *Alox5* and *Alox5ap* are expressed in human airway tuft cells (37) and mouse gastrointestinal tuft cells (38, 39). We found that, after infection, immature C57BL/6 mice showed increased lung cysLT levels and rare Alox5+ and Alox5ap+ cells in the airway epithelium with a triangular or bottle-like shape typical of tuft cells. *Pou2f3-/-* mice lacking tuft cells showed absent Alox5+ and Alox5ap+ airway epithelial cells and reduced lung cysLT levels, consistent with the notion that airway tuft cells are a significant source of lung cysLTs after RV infection.

Since montelukast is cysLT receptor antagonist, it should act downstream of tuft cell Alox5 activation, blocking the action of cysLTs on immune cells. However, we found that montelukast-treated mice also showed a reduction in lung cysLT levels and Alox5ap+ epithelial cells. It has been shown previously that montelukast treatment decreases lung cysLT production after heterologous RSV infection (40) and ovalbumin sensitization and challenge (41). These data are consistent with previous work showing that aeroallergen-induced expansion of tuft cells is dependent on Ltc4s (25), indicating an autocrine role for cysLTs. Thus, it appears that montelukast also acts upstream of tuft cell Alox5 activation, reducing tuft cell cysLT production in a feed-forward manner.

While RV-infected *Pou2f3-/-* mice showed absent Alox5ap+ airway epithelial cells, Alox5ap+ round cells were present in the subepithelium, likely representing expression by mast cells, eosinophils or macrophages. However, based on the lung cysLT measurements in the *Pou2f3*-/- mice, they are making a small amount of cysLTs compared to the tuft cells. This could be due to the location of the tuft cells in the airway epithelium, where they are more likely to contact the virus and initiate a CysLT response. This production was completely blocked in *Alox5*-/- mice. Finally, as we found previously (14), untreated mice showed no Alox5ap+ epithelial cells, consistent with the scarcity of tuft cells in the intrapulmonary airways.

Besides CysLTs, immunomodulatory tuft cells produce the innate cytokines IL-25 and TSLP (42). Consistent with this, RV-infected *Pou2f3-/-* mice lacking tuft cells also showed reduced mucous metaplasia, type 2 cytokine expression and airways responsiveness. To determine the requirement of cysLTs for the asthma phenotype, we also infected *Alox5*-/- and montelukast-treated mice. *Alox5*-/- and montelukast-treated mice also showed reduced mucous metaplasia, type 2 cytokine expression and airways responsiveness, suggesting that lung cysLTs are required for development of the asthma phenotype after RV infection. While montelukast, a cystLT receptor antagonist, has been found effective in improving asthma control in both pediatric and adult patients with chronic asthma (43, 44), the contribution of cysLTs in the development of viral-induced airways disease has not been well-studied. Montelukast suppressed double-stranded RNA-induced airway inflammation in mice with ovalbumin-induced allergic airways disease (45). CysLTs are increased in respiratory secretions from infants with acute viral bronchiolitis, suggesting a possible role of cysLTs in the pathogenesis of the disease (46). We similarly found increased *Alox5* and *Alox5ap* transcripts in infants with RV bronchiolitis. However, two randomized controlled trials examining the effect of montelukast on clinical course in acute bronchiolitis showed mixed effects, with one showing no effect (47) and one showing reduced length of stay (48).

We have not tested whether infection with RSV induces an asthma phenotype and tuft cell expansion in immature mice. Infection of 7 day-old mice with RSV causes severe structural alterations in lung development including the loss of alveoli (49), a phenotype which is drastically different from our model in which no change in the size of alveolar spaces is seen (unpublished data). In addition, it has been shown that tuft cells develop in the distal lung after severe influenza injury (50).

In conclusion, we conclude that tuft cell-derived cysLTs are required for mucous metaplasia, type 2 inflammation and airways hyperresponsiveness in immature mice exposed to heterologous viral infection. Further studies are needed to determine the importance of this pathway for viral-induced asthma development and exacerbations.

## Data Availability

The transcriptomic data presented in the study are deposited in the GEO repository, accession number GSE311749.
