# Supplementary material for: Tuft cell cysteinyl leukotrienes are necessary for rhinovirus-induced mucus metaplasia, type 2 inflammation and airway hyperresponsiveness in immature mice
Source: Front Immunol. 2026 Jan 7;16:1709008. doi: 10.3389/fimmu.2025.1709008 (PMC12819791; doi:10.3389/fimmu.2025.1709008)

Supplementary Material

**Tuft Cell Cysteinyl Leukotrienes Are Necessary for Rhinovirus-Induced Mucus Metaplasia, Type 2 inflammation and Airway Hyperresponsiveness in Immature Mice**

De’Jana T. Parker, J. Kelley Bentley, Jing Lei, Baljeet Domala, Hannah L. Briggs, Shilpi Singh, Yiran Li, M. Claire Reiner, Derek A. Flores, Alex L. Sliwicki, Heidi R. Flori and Marc B. Hershenson

**Figure. Effect of sex on RV-induced Muc5ac mRNA expression and airway methacholine responsiveness.** C57Bl/6 mice were inoculated with HeLa cell lysate or RV-A1B on day 6 of life and HeLa cell lysate or RV-A2 on day 13 of life. Lung Muc5ac mRNA was measured by qPCR. Expression relative to GAPDH was calculated using the 2–∆∆Ct method. Airways responsiveness was measured in anesthetized, tracheotomized mice using a Buxco FinePointe operating system. Airway responsiveness was assessed by measuring changes in total respiratory system resistance in response to nebulized methacholine.


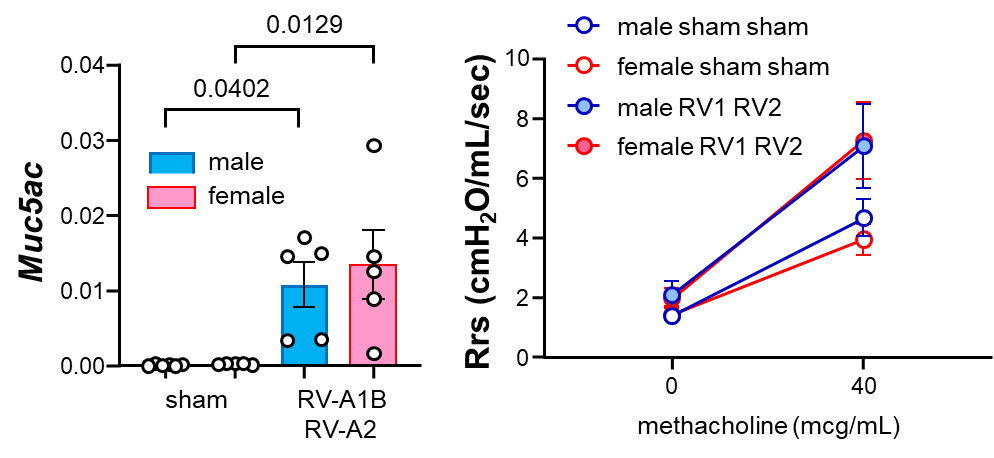

Supplement: Supplementary file 1 [file SupplementaryFile1.docx]
